# Cognitive dysfunction in young subjects with periodontal disease

**DOI:** 10.1007/s10072-021-05115-3

**Published:** 2021-02-19

**Authors:** Simona I. Hategan, Sabrina A. Kamer, Ronald G. Craig, Cosmin Sinescu, Mony J. de Leon, Dragos C. Jianu, Catalin Marian, Bianca I. Bora, Traian-Flavius Dan, Claudiu D. Birdac, Anca Marcu, Angela R. Kamer, Meda Lavinia Negrutiu

**Affiliations:** 1 Department of Prosthodontics, Faculty of Dentistry, University of Medicine and Pharmacy “Victor Babes” Timisoara, Bd. Revolutiei 1989, Nr.9, 300070 Timisoara, Romania; 2 Department of Biochemistry and Pharmacology, University of Medicine and Pharmacy “Victor Babes” Timisoara, Piata Eftimie Murgu, Nr 2, 300041 Timişoara, Romania; 3 Department of Periodontology and Implant Dentistry, College of Dentistry, New York University, 345 East 24th Street, New York, NY 10010 USA; 4 Department of Molecular Pathobiology, College of Dentistry, New York University, 345 East 24th Street, New York, NY 10010 USA; 5 Department of Prostheses Technology and Dental Materials, Faculty of Dentistry, University of Medicine and Pharmacy “Victor Babes” Timisoara, Bd. Revolutiei 1989, Nr.9, sc.C, et.IV, 300070 Timisoara, Romania; 6 Department of Radiology, Brain Health Imaging Institute, Weill Cornell Medicine, New York, NY 10019 USA; 7 Department of Neurology – Neurosciences, University of Medicine and Pharmacy “Victor Babes” Timisoara, Eftimie Murgu st., no.2, 300041, Timisoara, Romania; 8 The Centre for Cognitive Research in Neuropsychiatric Pathology (NeuroPsy-Cog), University of Medicine and Pharmacy “Victor Babes” Timisoara, Eftimie Murgu st.,no.2, 300041, Timisoara, Romania; 9 First Dept. of Neurology, Clinical County Emergency Hospital, Timisoara, Liviu Rebreanu Ave., no 156, 300723, Timisoara, Romania

**Keywords:** periodontal disease, Alzheimer's disease, episodic memory, neuropsychological assessment, salivary cytokines, cognitive dysfunction

## Abstract

**Background:**

Periodontal disease is an inflammatory, dysbiotic condition. Studies have shown that in the elderly, periodontal disease was associated with cognitive dysfunction and Alzheimer’s disease.

**Objective:**

To investigate whether young healthy subjects with periodontal disease have lower cognition compared to those without periodontal disease. The salivary cytokines (IL-1β, TNF-α) levels in relation to cognition were also tested.

**Methods:**

In a monocenter, cross-sectional study, forty subjects [mean age (SD) = 34 (5) and 48% female] from western Romania were classified into periodontal disease conditions using radiographic assessment: 10 subjects had aggressive periodontitis (AGG_P), 20 chronic mild-moderate periodontitis (CR_P), and 10 no periodontitis (NL_P). Neuropsychological assessment performed by standardized neurologists and psychologist included Rey Auditory Verbal Learning Test (RAVLT), Montreal Cognitive Assessment test (MOCA), Mini-Mental State Examination (MMSE), and Prague tests. Salivary cytokines levels were determined by ELISA.

**Results:**

RAVLT and MOCA delayed recall scores were lower in AGG_P group compared to NL_P and CR_P. The learning curve was also different with subjects with AGG_P showing reduced learning performance. Contrary to our hypothesis, salivary IL-1β associated with immediate but not delayed cognitive scores.

**Conclusions:**

These results showed for the first time that subjects with AGG_P had cognitive dysfunction and IL-1β may play a role in this process.

**Supplementary Information:**

The online version contains supplementary material available at 10.1007/s10072-021-05115-3.

Worldwide, approximately 50 million people have dementia among which 50–60% are diagnosed with Alzheimer’s disease (AD) (World Health Organization). It is estimated these numbers will almost double by 2030 and triple by 2050. Delaying the onset of AD by only 2 years could reduce the number of AD cases in 50 years by 2 million. These predictions underscore the importance of identifying modifiable risk factors earlier in life.

Recent animal and epidemiological studies suggested that peripheral inflammation and dysbiotic conditions contributed to AD pathogenesis [1–5]. Periodontal disease (PerioD) is a peripheral inflammatory, dysbiotic condition affecting more than 10% and 50% of the young and older population, respectively [6]. It results from the interaction between the dysbiotic bacteria and the host immune response leading to structural damage to tissues surrounding affected teeth [7].

Epidemiological data of various designs also linked PerioD and AD and reported that measures of PerioD were associated with cognitive dysfunction, cognitive decline, dementia, and AD, with odds and hazard risk ratios in the mild to moderate range [8–10]. Our studies showed increased brain amyloid accumulation [11], and cognitive dysfunction [12] in elderly with measures of periodontal disease. Most studies, including ours, investigated these relationships in elderly. Only a few studies included young populations [9, 13], and therefore, it is unclear whether these relationships are also found in youth. Studying younger populations is significant for several reasons: AD pathology starts early in life [14] with a long preclinical phase; longer periodontal exposure increases the AD risk [15]; the young population are most likely to lack other comorbidities that would affect AD, and preventive measures could be implemented early. Periodontal disease can occur in the young population in the form of chronic periodontitis or aggressive periodontitis. Aggressive periodontal disease is especially destructive and has significant local and systemic inflammation [16, 17]. By definition, aggressive periodontitis is found in young systemically healthy people, and its prevalence could be higher in some populations [18]. Therefore, this population would be ideal to investigate the role of periodontal disease in AD [15]. Episodic memory is one of the first memory domains to be impaired in AD and can be detected years before AD diagnosis [19, 20]. In addition, episodic memory associated with AD pathology in preclinical and prodromal stages [21, 22]. We hypothesized that young subjects with periodontal disease would have impaired episodic memory compared to controls. We also hypothesized that salivary proinflammatory molecules IL-1β and TNF-α would inversely correlate with delayed memory.

## Methods and materials

This was a monocenter, cross-sectional comparative study of 3 clinical groups of young medically healthy subjects from the western region of Romania. The subjects were derived from a pool of 149 subjects that participated in a previous retrospective study [18]. These subjects presented to the Prosthodontics Department of the Faculty of Dental Medicine, Victor Babeş University of Medicine and Pharmacy, Timisoara, for comprehensive dental treatment. This study was approved by the University Ethics Committee. Informed consent was reviewed and signed by all subjects (No27/2017). Sixty subjects were asked to participate in the “cognitive study.” Among them, 40 subjects agreed and were recruited: 10 with aggressive periodontitis (AGG_P), 20 with chronic mild-moderate periodontitis (CR_P), and 10 with no signs of periodontitis (NL_P). In addition to fulfilling the inclusion and exclusion criteria described below, subjects were required to agree to a neuropsychological evaluation and saliva collection. Diagnosis of periodontal conditions was done by two calibrated periodontists both with more than 20 years of clinical and research experience using panoramic radiographs as we previously published [18]. Radiographic images were also used to assess caries, tooth number, endodontic treatments, and periapical pathology [18].

### Inclusion/exclusion criteria

Included subjects were required to be fluent in Romanian and aged <45 years. Excluded subjects were those with significant medical history or conditions including diabetes, uncontrolled hypertension, head trauma with loss of consciousness, any neurodegenerative disease, chronic depression, past or current drug use, and taking anti-inflammatory medications for chronic conditions (i.e., NSAIDS, anti-TNFα). Subjects taking antibiotics or having periodontal treatment ≤3 months prior to entering the study were also excluded. The standardized examiner performed an interview to collect data on demographics (age, gender, education), history of systemic conditions, smoking, history of drugs, and current medications. Data on education was collected at 3 levels: high school graduate (12 years) (HSE), trade school graduate TSE (high school + trade school; 13–15 years), and higher education UE (high school + university; >15 years). Two subjects reporting 10 years of education were included in the first level.

### Outcome measures

The primary outcome measure was delayed recall memory tested by the Rey Auditory Verbal Learning Test (RAVLT). Secondary outcomes were immediate memory and learning assessed by Rey Auditory Verbal Learning Test (RAVLT). In addition, the Montreal Cognitive Assessment test (MOCA), Mini-Mental State Examination (MMSE), and Prague tests were also used [23].

### Clinical assessments

Neurological and medical examinations were performed in the Neurology Department, “Victor Babes” University of Medicine and Pharmacy, Clinical County Emergency Hospital by the Clinical Neurology Specialists and consisted of medical assessment, neurological exam, and neuropsychological assessment. The medical assessment included medical history review, blood pressure (BP), and heart rate measurement. Elevated blood pressure was defined if SBP≥140 or DBP≥90 mmHg. A full review of systems was performed with emphasis on neurological or related symptoms. The neurological assessment was quantified by the National Institutes of Health Stroke Scale (NIHSS-Romanian version) and was normal in all subjects (NIHSS scores=0). We observed no gait disturbances, no motor deficits, or meningeal signs. Subjects had normal tone in all four extremities, and tendon and plantar reflexes were normal and symmetrical. The sensation was intact to light touch, pinprick, proprioception, vibration, and temperature throughout; cranial nerves tests were normal, and the speech was fluent, with no errors in comprehension or repetition.

### Neuropsychological assessment

Neuropsychological assessments were performed by a clinical psychologist using RAVLT, MOCA, MMSE, and Prague tests. Romanian translations of each test were used [24–28].

**Fig. 1:**
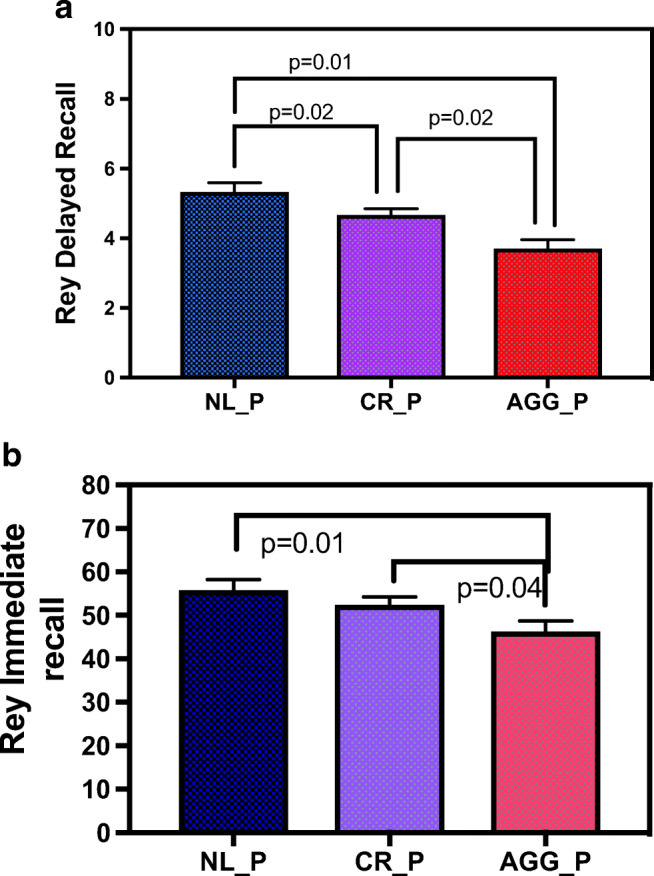
**a** RAVLT Delayed Recall among groups. ANOVA showed that there was a significant lower RAVLT delayed recall scores in AGG_P group compared to NL_P and CR_P groups (*p*=0.01 and *p*=0.02) and in CR_P compared to NL_P group (*p*=0.02). NL_P, no PerioD; CR_P, mild to moderate chronic PerioD; AGG_P, aggressive PerioD. **b** RAVLT Immediate recall among groups. ANOVA for RAVLT immediate recall showed significant difference in the periodontal groups: AGG_P group had significantly lower scores compared to NL_P group (*p*=0.01 and *p*=0.04). NL_P, no PerioD; CR_P, mild to moderate chronic PerioD; AGG_P, aggressive PerioD

- The RAVLT consisted of 6 learning trials during which the same 15-word list was read out loud. Immediately after each of the first 5 trials, the subject was asked to recall as many words as he/she could [27–29]. After 30 min, trial 6 was performed in which the subject was asked to recall as many words as he/she could from the initial list. As distraction, the psychologist conversed with the subject. Results were reported as scores for each of the different domains measured by the RAVLT. Delayed memory was defined as the score of trial 6 and ranged from 0 to 15. Immediate memory was defined as the sum of the scores from trials 1 to 5 and ranged 0–75. Learning was defined as the score of trial 5 minus trial 1. Forgetting was calculated as the scores of trial 5 minus trial 6. Percent forgetting was the forgetting score divided by the trial 5 score.
- MOCA consisted of both verbal and pencil/paper tasks assessing overall cognitive function and performance in areas of visuospatial/executive function, naming, memory, attention, language, abstraction, delayed recall, and orientation with a maximum score of 30 [25, 26]. As reported, MOCA scores were adjusted for education by adding 1 point to subjects with 12 or less years of education.
- MMSE consisted of a 30-point verbal and pencil-and-paper questionnaire [25]. The test concentrated on the assessment of orientation, attention and calculation, recall, language, and ability to follow simple commands.
- PRAGUE test was developed by the Psycho-technical Institute in Prague to assess distributive attention. The subject was presented with a 10×10 matrix and 4 columns with a list of 25 numbers in each column (Figure 1S). Each cell of the matrix had 2 numbers: one number written in bold while below, the second number written in smaller font. The subject was asked to match the number in the column to the corresponding number in the matrix and write down the smaller sized number associated with that specific cell. The test was carried out in a standard time of 16 min divided into sequences of 4 min for each column, with a 1-min pause between columns. The sum of all matched numbers represented the score and ranged from 0 to 100.

### Hamilton rating scale for depression (HRSD17)

A 17-item version of the HRSD (HRSD17) test was used to evaluate depression as a possible cause of cognitive decline [30].

### Saliva collection and cytokine assessment

Saliva collection and processing were done as published [31]. Salivary stimulation was achieved by chewing unflavored chew paraffin wax pellets (Glee Gum, Verve Inc., Providence, RI). Saliva was stored at −80°C until cytokine assays were performed. Salivary interleukin-1 (IL-1β) and tumor necrosis factor-α (TNF-α) levels were assessed using human IL-1β ELISA kit (Invitrogen, Thermo Fisher Scientific, CA, USA) and Human TNF-α Ultrasensitive ELISA kit (Invitrogen, Thermo Fisher Scientific, CA, USA) using the manufacturer’s protocol. The absorbance was read spectrophotometrically at 450 nm using a GloMax Discover instrument v3.0. (Promega Corp, WI, USA). Using the standard equation curves, saliva IL-1β and TNF-α concentrations were determined.

### Statistical methods

Statistical analyses were performed using IBM SPSS (v26, IBM Corp., Armonk, NY). Continuous data are presented as means and standard deviation (SD) and categorical data as percentages. Group differences for continuous variables were tested by ANOVA and Kruskal-Wallis *H* test, while for categorical variables, Chi-square or Fisher’s exact tests were used. Normality was tested by Kolmogorov-Smirnov, and log transformation was used to normalize the distributions for salivary IL-1β and TNF-α. To determine group differences for repeated variables, repeated measures ANOVA was done using the Greenhouse-Geisser test. The following covariates were tested in the initial models: age, gender, educational level, smoking, and the dental variables (carries, periapical lesions, crowns, roots). Since none of them were significant, they were dropped from the final model. Correlations and linear models were used to assess the cognitive relationship with cytokines.

## Results

Table 1 shows subject characteristics. All subjects were systemically healthy, and mean age was 34 (SD=5). Subjects were overall well educated, only 14% were smokers, and gender was equally distributed. When we compared the periodontal groups, there were no differences in age, gender, smoking, BP, dental lesions, or depression scores. We found differences in tooth number among the groups (*p*=0.01) and the percentage of subjects with only high school education (*p*=0.01).

**Table 1:** Characteristics of the study population

N	Total	NL	CR	AGG	*p* value			
	40	10	20	10	NL/CR/AGG	NL/CR	NL/AGG	CR/AGG
Demographics								
Age [mean (SD)]	33.67(5.34)	31.10 (5.38)	34.45 (5.06)	34.70 (5.52)	0.21	0.11	0.13	0.90
Gender (*n* (%))					0.95	0.80	1.00	0.80
Male	21 (51%)	5 (50%)	11 (55%)	5 (50%)				
Female	19 (48%)	5 (50%)	9 (45%)	5 (50%)				
Education level (*n* (%))					0.01	0.44	0.01	0.01
High school	9 (22.5%)	0 (0%)	2 (10%)	7 (70%)				
Trade education	8 (20%)	2 (20%	6 (30%)	0 (0%)				
University education	23 (57.5%)	8 (80%)	12 (60%)	3 (30%)				
Dental characteristics (mean (SD))								
Num teeth	25.85 (4.19)	28.20 (2.15)	26.55 (3.59)	22.10 (4.63)	0.01	0.22	0.01	0.02
Implants	0.23 (0.73)	0.10 (0.32)	0.40 (1.00)	0.00 (0.00)	0.28	0.65	0.74	0.40
Root number	0.48 (1.28)	0.50 (1.58)	0.10 (0.31)	1.20 (1.87)	0.10	0.98	0.32	0.16
Carious lesions	3.55 (2.85)	3.00 (2.49)	3.05 (2.19)	5.10 (3.90)	0.35	0.78	0.25	0.20
Crowns	3.00 (4.88)	1.90 (4.04)	3.65 (4.99)	2.80 (5.67)	0.41	0.25	0.91	0.42
Root canal	2.33 (2.28)	1.40 (1.58)	3.15 (2.58)	1.60 (1.71)	0.08	0.07	0.91	0.09
Fillings	6.23 (3.81)	5.50 (3.60)	7.60 (3.87)	4.20 (2.97)	0.07	0.23	0.48	0.02
Periapical lesions	0.70 (1.16)	0.20 (0.63)	0.75 (0.79)	1.10 (1.91)	0.13	0.07	0.20	0.85
Smoking (*n* (%))					0.90	0.78	0.64	0.79
Yes	14 (35%)	3 (30%)	7 (35%)	4 (40%)				
No	26 (65%)	7 (70%)	13 (65%)	6 (60%)				
Blood pressure (*n* (%))					0.14	0.56	0.07	0.11
Normal	26 (65%)	8 (80%)	14 (70%)	4 (40%)				
Elevated	14 (35%)	2 (20%)	6 (30%)	6 (60%)				
HRSD17 (mean (SD))	3.30 (1.91)	2.80 (2.30)	3.40 (1.60)	3.60 (2.22)	0.52	0.31	0.39	0.85

*NL* no periodontitis, *CR* mild/moderate chronic periodontitis, *AGG* aggressive periodontitis, *HRSD17* 17-item Hamilton rating scale for depression

*p values provided are non-adjusted for multiple comparisons

### Delayed recall and immediate recall scores were lower in subjects with periodontitis

In 1-way ANOVA (Fig. 1a and Table 2), we found that there was a significant difference in RAVLT delayed recall scores among the periodontal groups [Means (SD): NL=9.70 (1.89) vs. CR_P=7.90 (2.10) vs. AGG_P=6.10 (1.66); F(2,37)=8.51, p=0.001]. RAVLT delayed recall scores were lower in AGG_P group compared to NL_P and CR_P (*p*=0.01 and *p*=0.02, respectively) and in CR_P compared to NL_P group (*p*=0.02). Consistent with these results, MOCA delayed recall scores were also lower in the AGG_P group [Mean (SD)= 2.40 (1.35)] compared to NL_P [Mean (SD)= 3.60 (1.08), *p*=0.05] and CP_P [Mean (SD)= 3.40 (0.94), *p*=0.04]. Since the age and educational levels were not significant in any of the models, they were not included in the models. As Fig. 1b shows, there was also a significant difference in RAVLT immediate recall scores among the periodontal groups [Means (SD): NL=55.80 (6.14) vs. CR_P=52.50 (8.10) vs. AGG_P=46.30 (7.73); F(2,37)=4.11, *p*=0.02]. Immediate recall scores were lower in AGG_P group compared to NL_P and CR_P (*p*=0.01 and *p*=0.04). RAVLT Percent forgetting, MOCA visuospatial, and Prague tests were also significant among groups but not any other tests as shown in Table 2.

**Table 2:** Cognitive scores in relation to the periodontal groups

N	Total	NL	CR	AGG	*p* value			
	40	10	20	10	NL/CR/AGG	NL/CR	NL/AGG	CR/AGG
RAVLT (mean (SD))								
Delayed recall	7.90 (2.30)	9.70 (1.89)	7.90 (2.10)	6.10 (1.66)	0.01	0.02	0.01	0.02
Immediate recall	51.78 (8.16)	55.80 (6.14)	52.50 (8.10)	46.30 (7.73)	0.02	0.67	0.01	0.04
Learning	6.03 (1.94)	6.90 (1.37)	6.20 (2.01)	4.80 (1.81)	0.04	0.33	0.01	0.06
Forgetting	5.05 (1.55)	4.40 (1.58)	5.30 (1.69)	5.20 (1.14)	0.31	0.14	0.25	0.87
Percent forgetting	0.40 (0.13)	0.31 (0.11)	0.40 (0.13)	0.46 (0.09)	0.02	0.05	0.01	0.19
MOCA (mean (SD))								
Total	27.20 (2.02)	28.20 (1.40)	27.55 (1.57)	25.50 (2.42)	0.01	0.35	0.01	0.01
Raw	26.98 (2.19)	28.20 (1.40)	27.45 (1.67)	24.80 (2.35)	0.01	0.29	0.01	0.01
Delayed recall	3.20 (1.16)	3.60 (1.08)	3.40 (0.94)	2.40 (1.35)	0.06	0.65	0.05	0.04
Visuospatial	4.60 (0.67)	4.90 (0.32)	4.70 (0.57)	4.10 (0.88)	0.03	0.50	0.04	0.08
Naming	2.98 (0.16)	3.00 (0.00)	3.00 (0.00)	2.90 (0.32)	0.22	1.00	0.74	0.68
Attention	5.83 (0.45)	5.90 (0.32)	5.85 (0.37)	5.70 (0.68)	0.78	0.85	0.68	0.78
Language	2.60 (0.63)	2.80 (0.42)	2.65 (0.67)	2.30 (0.68)	0.13	0.78	0.12	0.17
Abstraction	1.93 (0.42)	2.00 (0.00)	2.00 (0.32)	1.70 (0.68)	0.21	1.00	0.48	0.40
Orientation	5.90 (0.38)	6.00 (0.00)	5.95 (0.22)	5.70 (0.68)	0.20	0.85	0.48	0.50
MMSE (mean (SD))								
Total	28.73 (1.22)	29.20 (0.79)	28.75 (0.97)	28.20 (1.81)	0.19	0.34	0.07	0.24
Delayed recall	2.68 (0.53)	2.70 (0.48)	2.70 (0.57)	2.60 (0.52)	0.77	0.88	0.74	0.59
Orientation	9.98 (0.16)	10.00 (0.00)	10.00 (0.00)	9.90 (0.32)	0.22	1.00	0.74	0.68
Attention	4.30 (0.91)	4.50 (0.71)	4.40 (0.60)	3.90 (1.45)	0.69	0.62	0.48	0.68
Language	8.00 (0.00)	8.00 (0.00)	8.00 (0.00)	8.00 (0.00)	1.00	1.00	1.00	1.00
Reg/Rep	3.00 (0.00)	3.00 (0.00)	3.00 (0.00)	3.00 (0.00)	1.00	1.00	1.00	1.00
Visuospatial	0.85 (0.36)	1.00 (0.00)	0.80 (0.41)	0.80 (0.42)	0.33	0.39	0.48	1.00
PRAG (mean (SD))	71.72 (14.06)	82.50 (13.24)	69.30 (13.34)	65.80 (11.14)	0.01	0.01	0.01	0.49

*NL* no periodontitis, *CR* mild-moderate/chronic periodontitis, *AGG* aggressive periodontitis, *MMSE* Mini-Mental State Examination, *MOCA* Montreal Cognitive Assessment, *MOCA total* adjusted for education, *MOCA raw* original raw score, *RAVLT* Rey Auditory Verbal Learning Test, *PRAG* Prague test

### Learning curves differed among the periodontal groups

**Fig. 2:**
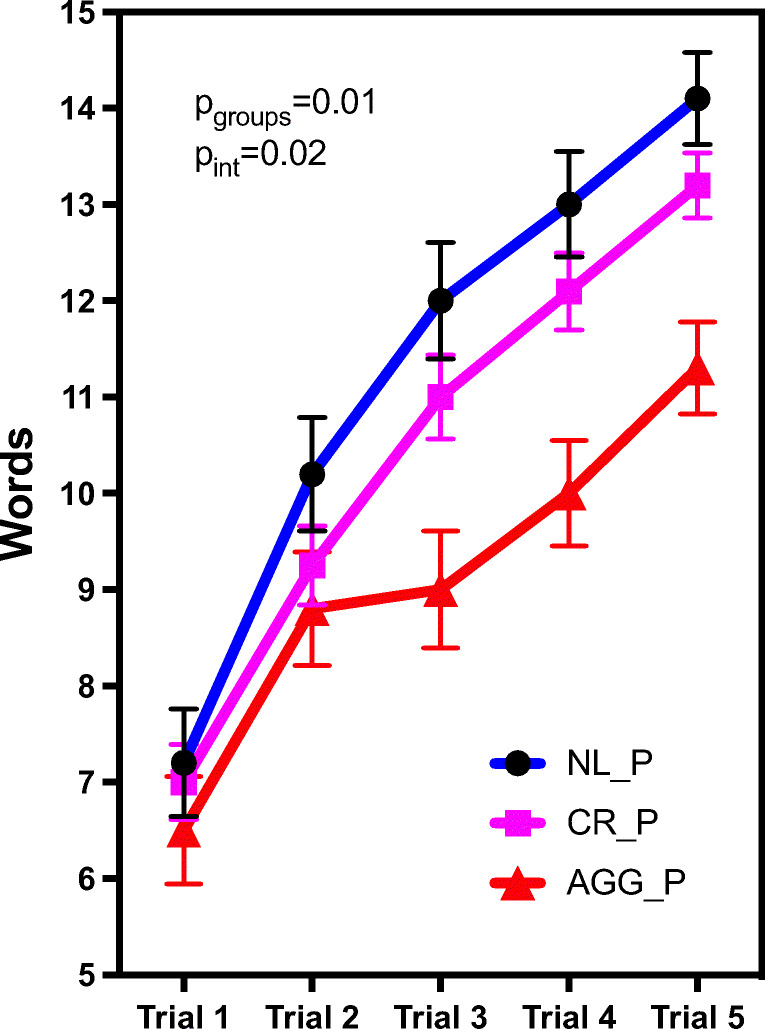
Learning performance over time (trials 1–5). ANOVA showed that learning performance differs among groups and AGG_P subjects had reduced number of words recalled, especially through trials 3 to 5, *p*=0.01. NL_P, no PerioD; CR_P, mild to moderate chronic PerioD; AGG_P, aggressive PerioD

A repeated measures ANOVA was run to determine the effect of periodontal groups on words recalled over time as assessed with trials 1–5. As shown in Fig. 2, there was a statistically significant interaction between periodontal groups on words recalled over time [F(6, 111)=2.61, *p*=0.02, partial *η*2=0.12]. Simple main effects showed that in trials 1 and 2, there was not a statistically significant difference among the periodontal groups [Means (SD): trial 1, NL_P=7.20 (1.15), CR_P=7.00 (2.15), AGG_P=6.50 (1.27); trial 2, (NL_P=10.20 (2.04), CR_P=9.25 (1.94), AGG_P=8.80 (1.40), *p*>0.05]. However, through trials 3 to 5, there was a significant difference between the words recalled between the AGG_P group and the other 2 groups (NL_P and CP_P) [Means (SD): trial 3, NL_P=12.00 (2.05), CR_P=11.0 (1.84), AGG_P=9.00 (2.05), *p*=0.01; trial 4, (NL_P=13.00 (1.15), CR_P=12.10 (2.00), AGG_P=10.00 (1.63), *p*=0.01; trial 5: (NL_P=14.10 (0.74), CR_P=13.20 (1.73), AGG_P=11.30 (1.64), *p*=0.01]. As shown in Fig. 2, the learning performance differed among groups, and subjects with aggressive periodontitis had reduced learning performance.

### Salivary IL-1β but not TNF-α associated with immediate cognitive scores

**Fig. 3:**
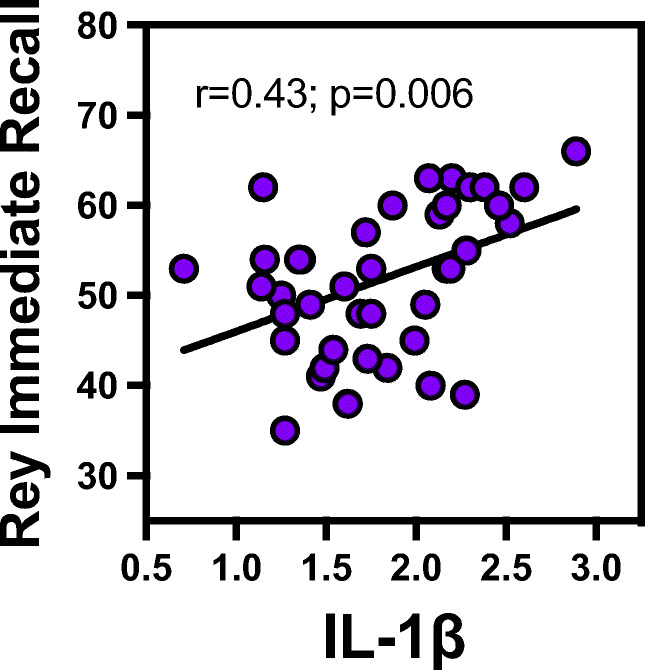
Correlation of IL-1β with immediate recall. Correlation and linear regression model showed that salivary IL-1β associated with Rey immediate recall (*r*=0.43, *p*=0.01)

In linear regression, salivary log IL-1β associated with Rey immediate recall *r*=0.43, *p*=0.01 (Fig. 3) while delayed recall did not (*r*=0.30, *p*=0.06). Salivary TNF-α did not associate with any cognitive tests.

## Discussion

Our study showed for the first time that among young systemically healthy subjects, those with AGG_P had impaired delayed episodic memory and learning rate compared to NL_P and CR_P. This conclusion was based on RAVLT and other cognitive tests showing significantly lower scores in AGG_P compared to NL_P. RAVLT delayed recall, percent forgetting, and Prague test were also lower in CR_P compared to NL_P. These results appear to be independent of age or education as both were not significant in any model. These results showed that periodontal disease may constitute a risk for cognitive impairment and this risk was most elevated in AGG_P. In addition, we found a significant positive correlation of salivary IL-1β and immediate recall scores suggesting a role in cognition.

Episodic memory is thought to be the first memory domain to be impaired in AD [32]. Studies showed that in addition to delayed recall, learning curves were also impaired in those with MCI compared to those with normal cognition [33]. These tests discriminated the most between cognitively normal and AD [34] and were predictors of early AD [33, 35]. Impairments in these cognitive tests have been associated with brain neurodegeneration and the lesions of AD. Immediate recall also depends on the learning ability and information coding, and these impairments have been associated with atrophy in frontal as well as temporal lobe [32], while delayed recall task was associated with the medial temporal area. Early memory impairment was found to associate with early AD with pathological findings localized in the mesial temporal lobes, especially in the hippocampal formation and entorhinal cortices [36, 37]. In addition to memory, attention assessed by Prague test was also compromised in both CR and AGG_P groups, and these results were consistent with our previous studies in elderly [12].

Our findings raised the possibility that in young subjects with periodontal disease, memory dysfunction is present, signs of brain abnormalities may exist, and increased risk of AD later in life is possible.

The difference in cognitive tests between NL_P and those with AGG_P was consistent across multiple cognitive tests. These results are not surprising as AGG_P is highly destructive and associates with more severe immune responses compared to CR_P. The microbial load is also higher and characterized by many pathogenic bacteria. The difference between those with CR_P and NL_P was not as consistent. This is likely due to less severe periodontal disease, less aggressive immune response, or less microbial burden. An additional reason could be the limited sample size. The cognitive tests for CR_P were slightly lower than those of NL_P, and therefore, a larger number could result in significance.

Proinflammatory cytokines such as IL*-*1β and TNF-α could contribute to neuroinflammation. However, they also have physiological roles [38]. IL*-*1β is required for proper learning and therefore immediate memory [39]. In our young population, higher IL-1β correlated with higher immediate memory. Their effects can also depend on timing, concentration, and duration of exposure [40]. We speculate that higher IL-1β facilitates cognition. On the other hand, we do not know the source of salivary IL*-*1β. It can be derived from the oral cavity or can be derived from systemic sources. Systemic sources are unlikely as these subjects are young and systemically healthy. It can also be derived from the brain. To untangle the role of oral cytokines in cognition and brain pathology, longitudinal studies are warranted with serial exams, and levels of IL-1β in saliva, blood, and CSF.

### Strengths and weaknesses

The strength of this study consisted of our relatively homogeneous population. All subjects were young and healthy. All medical and neuropsychological exams were performed by well-trained neurologists and a psychologist that were blind to the periodontal diagnosis. Equally, the periodontists classifying the periodontal conditions were blind to the neurological and cognitive findings.

There are several limitations related to our study that include the design, population characteristics, and sample size. Being cross-sectional, our study did not allow inference regarding causation. It was also possible that subjects with cognitive dysfunction had poorer oral hygiene and therefore poorer periodontal conditions. However, periodontal disease destruction results from the interplay between periodontal bacteria and host immune response, and this individual immune response plays a major role in AGG_P periodontitis. Although the number of subjects in this study was relatively small, statistically significant differences were found. Subsequent studies will use the data of the present study for power calculations in larger follow-up studies.

Education was defined by groups. Although it was not significant in any model, a study matching for years of education would be desirable.

An additional bias may be related to the participants themselves. Our sample was derived from people seeking prosthodontic treatment. However, this sample may be self-selected, thus introducing a potential bias. An additional weakness was the periodontal diagnosis which was done by X-ray as described in our previous publication (48). Clinical diagnosis with periodontal inflammatory measures done by standardized, calibrated personal would be needed.

In conclusion, we showed that young subjects with periodontal disease had lower cognition. We also showed that contrary to our prediction, proinflammatory cytokine IL-1β may be a facilitator of cognition. However, a larger study with control of modifiable variables (diagnostic criteria, time of diagnosis, and follow-up between periodontitis and cognitive decline, level of education, etc.) is needed. Continued investigation of modifiable variables in AD, such as periodontal disease, provides new directions for treatments and therapies which could considerably alter the future impact of AD.

## Supplementary information

ESM 1 (DOCX 1113 kb).

## Data Availability

Not applicable.
