# Supplementary material for: Cognitive dysfunction in young subjects with periodontal disease
Source: Neurol Sci. 2021 Feb 19;42(11):4511–9. doi: 10.1007/s10072-021-05115-3 (PMC8519837; doi:10.1007/s10072-021-05115-3)
Supplement: Supplementary file 1 — (DOCX 1113 kb). [file 10072_2021_5115_MOESM1_ESM.docx]

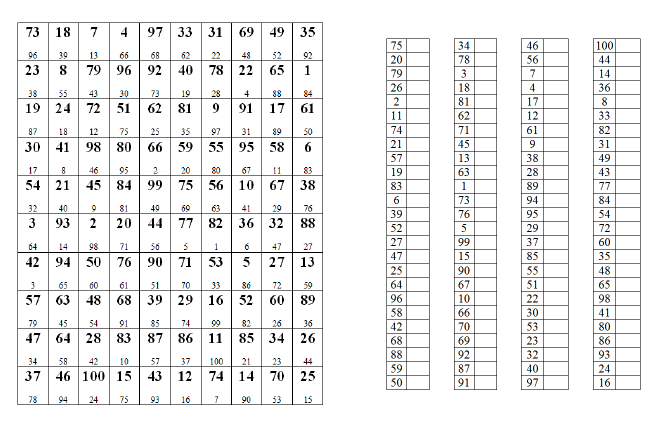


**Figure 1S.** Prague test administration. The subject is presented with a 10X10 matrix as shown in A and 4 separate test columns as shown in B. Each square of the matrix contains a pair of numbers: one in bold, large font and the second one below in small font. Each column in B contains a set of rectangles with a number and an empty field. The subject is tasked to identify the number in the rectangle of the column B among the bold numbers of the matrix and then writing the corresponding small font number in the empty rectangle of column B. For example, the first number in the first column is 75. This number can be identified in the 6^th^ column and 5^th^ row of the matrix. The corresponding number in small font of that cell is 69. Therefore, 69 is written in the empty column next to 75. The test is carried out in a standard time of 16 minutes divided into sequences of 4 minutes for each column, with a 1-minute pause between columns. The sum of all matched numbers represents the score.
